# The immune-body cytokine network defines a social architecture of cell interactions

**DOI:** 10.1186/1745-6150-1-32

**Published:** 2006-10-24

**Authors:** Ziv Frankenstein, Uri Alon, Irun R Cohen

**Affiliations:** 1 Department of Immunology, The Weizmann Institute of Science, Rehovot 76100, Israel; 2 Department of Molecular Cell Biology and Department of Physics of Complex Systems, The Weizmann Institute of Science, Rehovot 76100, Israel

## Abstract

**Background:**

Three networks of intercellular communication can be associated with cytokine secretion; one limited to cells of the immune system (immune cells), one limited to parenchymal cells of organs and tissues (body cells), and one involving interactions between immune and body cells (immune-body interface). These cytokine connections determine the inflammatory response to injury and subsequent healing as well as the biologic consequences of the adaptive immune response to antigens. We informatically probed the cytokine database to uncover the underlying network architecture of the three networks.

**Results:**

We now report that the three cytokine networks are among the densest of complex networks yet studied, and each features a characteristic profile of specific three-cell motifs. Some legitimate cytokine connections are shunned (anti-motifs). Certain immune cells can be paired by their input-output positions in a cytokine architecture tree of five tiers: macrophages (MΦ) and B cells (BC) comprise the first tier; the second tier is formed by T helper 1 (Th1) and T helper 2 (Th2) cells; the third tier includes dendritic cells (DC), mast cells (MAST), Natural Killer T cells (NK-T) and others; the fourth tier is formed by neutrophils (NEUT) and Natural Killer cells (NK); and the Cytotoxic T cell (CTL) stand alone as a fifth tier. The three-cell cytokine motif architecture of immune system cells places the immune system in a super-family that includes social networks and the World Wide Web. Body cells are less clearly stratified, although cells involved in wound healing and angiogenesis are most highly interconnected with immune cells.

**Conclusion:**

Cytokine network architecture creates an innate cell-communication platform that organizes the biologic outcome of antigen recognition and inflammation. Informatics sheds new light on immune-body systems organization.

**Reviewers:**

This article was reviewed by Neil Greenspan, Matthias von Herrath and Anne Cooke.

## Open peer review

Reviewed by Neil Greenspan, Matthias von Herrath and Anne Cooke. For the full reviews, please go to the Reviewers' comments section.

## Background

Until recently, the attention of immunology was focused primarily on the molecular and cellular mechanisms by which lymphocytes recognise specific antigens [1-3]. However, it has now become clear that the behaviour of the immune system is greatly influenced by signalling between interacting cells, including cells that do not directly recognise antigens [1-3]. Cytokines ("cell activators") are prominent among the innate signals that determine the biologic outcome of the adaptive immune response to specific antigens and the response to inflammatory stimuli generally [1-4]. This study analyzes informatically the cytokine network with the aim of uncovering its characteristic features: connection density, motifs and anti-motifs, distinct cell roles, and network super-family associations.

## Results

### Cytokine connectivity is exceptionally dense

Cytokine connections between and among immune and body cells (see Table 1) were obtained manually from two Internet databases: the Cytokines Online Pathfinder Encyclopedia (COPE)[5,6] and the Cytokine Reference – Online Database[7]. We transformed automatically the raw data into a network format designating cells as nodes and cytokine connections as edges (see additional file 1 for details). The computational and algorithmic tools presently available for network analysis did not allow us to study the particular cytokines that connect two or more cells, but only whether the connectivity is unidirectional – only one of the cells produces cytokines to which the other responds (designated by a single-headed arrow) – or bidirectional – the connected cells mutually respond to at least one of each other's cytokines (designated by arrowheads at both ends of an edge). This grouping of individual cytokines reduced 2461 individual edges to 418 composite edges connecting 29 nodes (immune and non-immune cells) in a global network that could be analyzed using existing algorithms (see additional files 1 and 2 for details). This global network was then divided into 3 component sub-networks (see additional file 2 for details): Immune sub-network – 111 edges connecting 14 nodes (immune cells only); Non-immune (body) sub-network – 84 edges connecting 15 nodes (non-immune cells only); and Interface sub-network – 223 edges connecting at least one immune cell with at least one body cell.

**Table 1:** Cytokines (edges) and cells (nodes) in the analysis

Cytokines (edges)	IFN-alpha	IL-5	IL-13	M-CSF
	IFN-beta	IL-6	IL-15	TGF-beta
	IFN-gamma	IL-7	IL-16	TNF-alpha
	IFN-kappa	IL-8	IL-18	TNF-beta
	IL-1	IL-9	IL-22	MIF
	IL-2	IL-10	IL-27	
	IL-3	IL-11	G-CSF	
	IL-4	IL-12	GM-CSF	
	Abbreviations used in Figures and Tables: IFN-Interferon; IL-Interleukin; CSF-Colony Stimulating Factor; TGF-Transforming Growth Factor; TNF-Tumor Necrosis Factor; MIF-Migration Inhibition Factor			
Immune cells (nodes)	MΦ-Macrophage/Monocyte	NK-T-Natural Killer T cell		
	NK-Natural Killer cell	DC-Dendritic cell		
	Th1-T helper 1	EOS-Eosinophil		
	Th2-T helper 2	BAS-Basophile		
	CTL-Cytotoxic T Cell	NEUT-Neutrophile		
	Tr1-T Regulatory 1	BC-B cell		
	DETC-Dendritic Epidermal T cell	MAST-Mast cell		

Body cells (nodes)	FIB-Fibroblast	OSTb-Osteoblast		
	EPIT-Epithelial cell	OSTc-Osteoclast		
	ENDO-Endothelial cell	ADIP-Adipocyte		
	PLAT-Platelet	SYNO-Synovial cell		
	CHON-Chondrocyte	REDc-Red Blood cell		
	NEUR-Neuronal cell	EPID-Epidermal cell		
	SMmus-Smooth muscle cell	MELA-Melanocyte		
	SKmus-Skeletal muscle cell			

**Figure 1:**
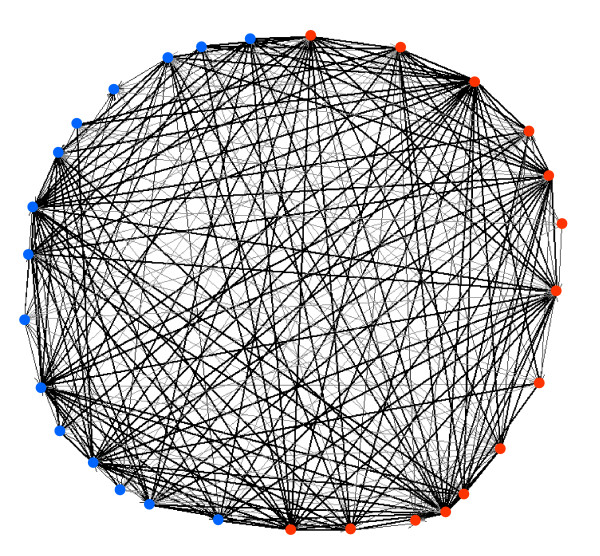
**The cytokine network**. Image of the global network of cytokine interactions between the 14 immune cells (red nodes) and the 15 non-immune body cells (blue nodes). The black edges represent mutual connections; the grey edges represent one-way connections.

Figure 1 illustrates the density (see Methods) of the global network of cytokine interactions between the 14 immune cells and the 15 non-immune body cells. Note that every cell is highly connected to other cells in mutual and one-way interactions.

The density for each of 113 different published networks was computed (see additional file 3) and the 30 densest are listed in Table 2. Note the following:

**Table 2:** The 30 densest published networks

**Network**		**N (nodes)**	**L (edges)**	**Density**	**Ref**.
Cytokines	Immune cells	14	111	0.61	-
Cytokines	Body cells	15	84	0.40	-
Social	*Student relationships	573	57029	0.35	29
Food-web	Skipwith Pond	25	189	0.32	14
Neural	Cat brain	52	820	0.31	21
Cytokines	Immune-body cell interface	28	223	0.30	-
Food-web	Coachella Valley	29	243	0.30	14
Food-web	Bridge Brook Lake	25	104	0.17	14
Neural	Monkey brain	71	746	0.15	21
Social	Sociology freshmen	28	110	0.15	14
Technological	*Airport of China	128	1165	0.14	27
Food-web	Little rock	92	984	0.12	14
Food-web	St Martin Island	42	205	0.12	14
Food-web	Freshwater	92	997	0.12	29
Technological	*Train routes	587	19603	0.11	29
Protein-structure	*Geometric model	53	136	0.10	14
Social	Leadership	32	96	0.10	14
Protein-structure	*Serine protease inhibitor	53	123	0.09	14
Food-web	Chesapeake Bay	31	67	0.07	14
Food-web	Ythan Estuary	83	391	0.06	14
Protein-structure	*Immunoglobulin	95	213	0.05	14
Protein-structure	*Oxidoreductase	99	212	0.04	14
Transcription-factor	Sea-urchin	45	83	0.04	14
Social	Inmates in prison	67	182	0.04	14
Food-web	Marine	135	598	0.03	29
Neural	C. elegans	280	2170	0.03	14
Transcription-factor	Drosophila	110	307	0.03	14
Information	*BA model	1000	9901	0.02	14
Metabolism	M. pneumoniae	178	470	0.02	28
Metabolism	A. pernix	204	588	0.01	28

*Undirected network

-This paper

The immune cytokine sub-network is the densest of networks: density score 0.61.

The non-immune (body cell) sub-network is second densest: density score 0.4.

Five networks exhibit density scores around 0.3: a social network relationship between students; two different food chain networks; a network of neurons in the cat brain; and the cytokine interface sub-network between immune system cells and non-immune body cells. The vast majority of the 113 networks we studied show densities well below 0.2 (see Table 2 and additional file 3): for example, C. elegans nervous system (score 0.03); drosophila transcription factors (score 0.03); E. coli metabolic networks (score 0.005) and transcription factors (score 0.003); yeast transcription factors (score 0.002); human protein interactions (score 0.004); drosophila protein interactions (score 0.0004); and the English Word-adjacency-text (score 0.0008). A recent study of synaptic connections between four-cell sets of pyramidal neurons in the rat visual cortex revealed a density of about 0.12[8]. Thus, cytokine connectivity among and between immune and body cells is remarkably dense compared to other known networks.

### Reciprocal connections

**Figure 2:**
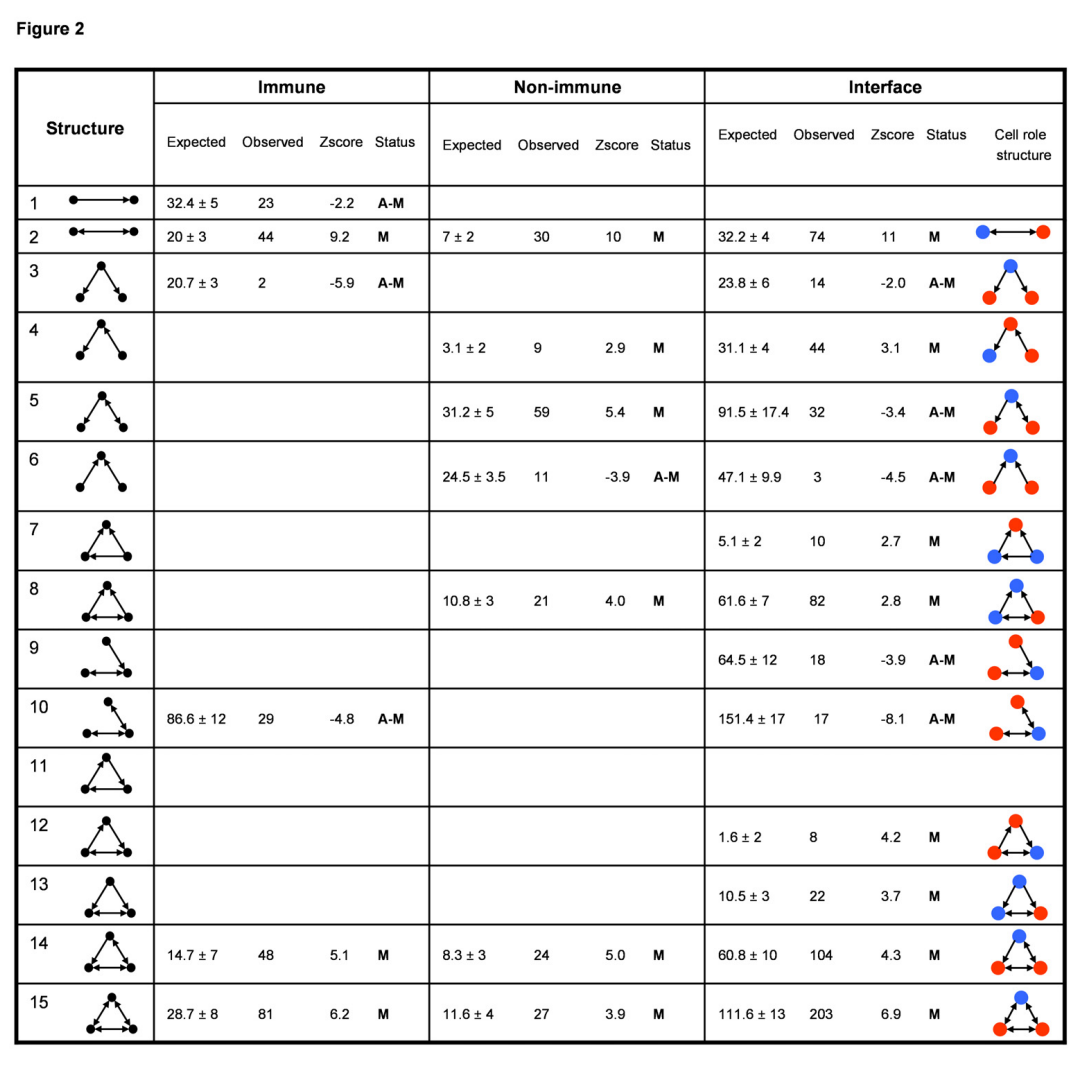
**Most significant Two and Three-node network motifs and anti-motifs**. The most significant motifs and anti-motifs (see Methods) in the two-node and three-node cytokine networks of immune cells, non-immune (body) cells, and immune-body interface. M (motif), A-M (anti-motif), red node (immune cell) and blue node (non-immune body cell).

Groups of nodes (pairs of cells; three-cell groups; and so forth) in a network may be organized in motifs (statistically more frequent than expected), anti-motifs (statistically less frequent than expected) or non-motifs (the expected frequency). The profiles of motifs and anti-motifs manifest the functional preferences and repudiations of the particular network. Figure 2 lists the most significant motifs and anti-motifs (see Methods) in the two-node and three-node cytokine networks of immune cells, non-immune (body) cells, and immune-body interface. Note that one-way connections between any two immune cells (Structure 1) are an anti-motif; this connection appears significantly less than expected in the immune sub-network. One-way connections between non-immune cells and at the immune-body interface appear neither more nor less than expected; they are non-motifs. However, reciprocally connected pairs of cells (Structure 2) are motifs in each of the three sub-networks – immune, non-immune and interface.

With regard to three-cell cytokine connections, the immune sub-network features two anti-motifs – Structures 3 and 10. The immune sub-network also features two motifs – the *semi-clique *and the *clique *(Structures 14 and 15) – the motifs richest in mutual interactions. Others have also noted these structures in the immune system[9], and these motifs appear in visual-cortex neuron networks[8].

The non-immune sub-network contains 5 three-cell motifs: Structures 4, 5 and 8 are motifs, in addition to Structures 14 and 15. Structure 6 is an anti-motif.

The immune-body interface is the richest in three-cell motifs and anti-motifs: Structures 4, 7, 8, 12, 13, 14 and 15 are motifs, and Structures 3, 5, 6, 9 and 10 are anti-motifs. We have colour-coded immune cells (red) and body cells (blue) to highlight the different three-cell network roles of immune cells and body cells. Note that the 5 interface anti-motifs express a common feature: two unconnected immune cells are not likely to interact with one body cell. However, two immune cells can interact with a body cell if they themselves are connected: Note that Structures 4, 12, 14 and 15, which link two connected immune cells and one body cell, are motifs. Moreover, two body cells may connect to one immune cell if the body cells themselves are connected (see motif Structures 7, 8 and 13). It thus appears that immune and immune-body interface cytokine networks are enriched for highly connected and reciprocally connected immune cells. Immune cells would appear to work collectively in connecting to the body.

### Immune and body cell network structures

**Figure 3:**
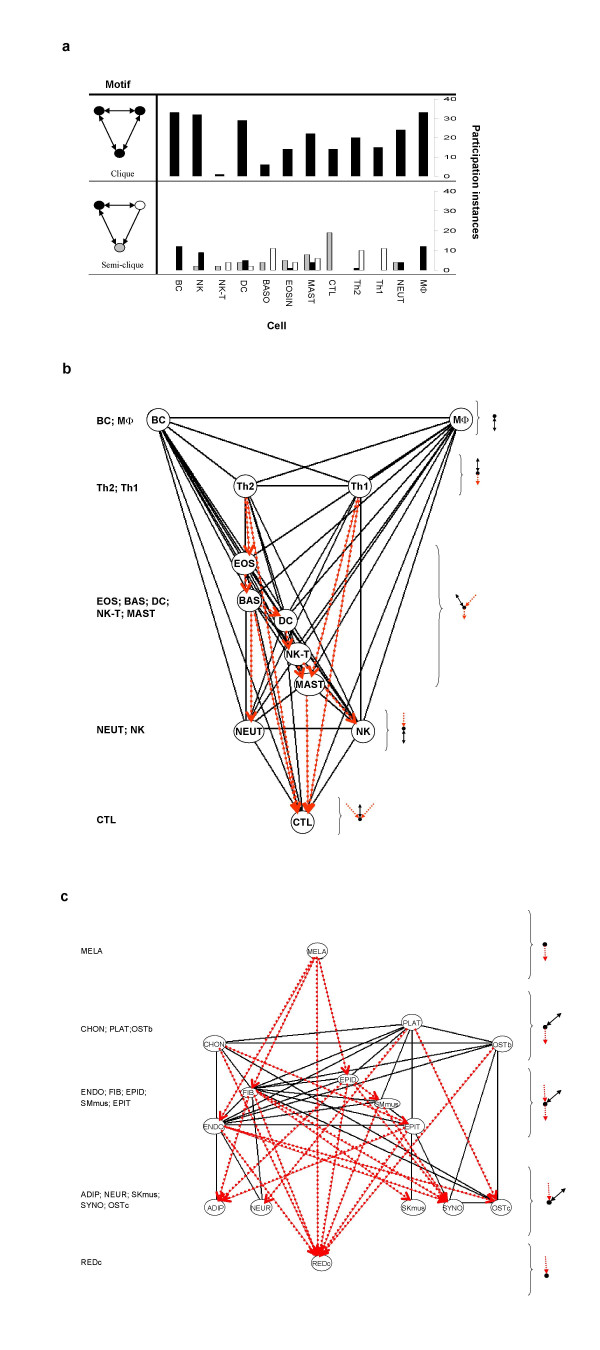
**Immune and body three-cell network structures**. **a**, The numbers of instances in which each type of immune cell participates in the *clique *and *semi-clique *motifs are shown. The three cells in the *clique *triad (upper panel) have no distinction between the connectivities of the participating cells (coded black bars). In contrast, each of the three nodes in the *semi-clique *(lower panel) has a distinct connectivity role (coded white, black or grey). **b**, Social cytokine architecture of immune cells. Combining all the instances of the clique and semi-clique triads occurring in the immune sub-network generates a five-tiered social hierarchy of immune cells by their mutual (black, full) and one-way (red, dashed) connectivities to the other nodes. **c**, Social cytokine architecture of body cells. Combining all the instances of the triads occurring in the body sub-network by their mutual (black, full) and one-way (red, dashed) connectivities to the other nodes generates a connectivity structure that appears quite different from that of the immune cells; see text for discussion.

We analyzed whether particular immune cell types could be assigned to particular nodes in the cytokine networks. Figure 3a shows the numbers of different cytokine arrangements in which each type of immune cell participates in the *clique *and in each of the three *semi-clique *cell roles. The three cells in the *clique *triad are each mutually connected to the other partner cells, so there can be no distinction between the possible connectivity roles of the participating cells. The *semi-clique *triad, in contrast, features cells with three different roles[10]: A cell may be mutually connected to the other two (Black node); a cell may be mutually connected to one cell and send a one-way output to another cell (White node); and a cell may be mutually connected to one cell and receive a one-way input from another cell (Grey node).

Figure 3a, lower panel, shows that certain immune cell types play characteristic roles in the *semi-clique*: Macrophages and B cells appear only in the role of the reciprocally connected node (Black node). Th1 and Th2 T cells appear mostly in the role of output cells in the *semi-clique *(White node). The CTL is notable among immune cells; although the CTL is mutually connected in the *clique*, its role in the *semi-clique *is exclusively that of the cell receiving one-way input (Gray node). Other cell types play mixed roles (note that T Regulatory 1 (Tr1) and Dendritic Epidermal T cell (DETC) are not included in Figure 3 due to insufficient data).

The mutual and one-way cytokine connectivities in the *clique *and *semi-clique *motif data (Figure 3a) can be combined to form a cytokine connection architecture for immune cell types (Figure 3b). This way of visualising cytokine connectivity reveals the following five-tiered system structure: The macrophage and the B cell form a pair in the first tier; these cells are reciprocally connected by cytokines to all the other cells of the immune system. All immune cells participate in mutual connections, but only the macrophage and the B cell have none but mutual connections. Th1 and Th2 T cells also can be paired in a second tier; this pair of T cells is mutually connected to the other cells, but is unique in sending out one-way cytokine signals to a variety of other cell types. One might reason that such an arrangement suits the regulatory function of this pair of T cells [1-3]. Alone in the fifth tier of immune cell society is the CTL [1-3]; this is the one cell type that receives multiple one-way inputs. The CTL functions to kill other cells, and multiple inputs could be imagined to help control the killing. Neutrophils and NK cells receive more limited one-way inputs, and constitute a fourth tier; unlike the CTL, these innate effector cells do not recognise antigens [1-3]. In the middle tier of the cytokine social structure are the remaining immune cells; these cells both receive one-way inputs and send one-way outputs.

The non-immune and interface sub-networks allow many different cell types to appear in the various nodes present in three-cell connections, and these sub-networks do not show the clear five-tiered hierarchy of the immune cells. Nevertheless, the network architecture of the non-immune body cells does show some interesting features (Figure 3c): fibroblasts (FIB), epithelial cells (EPIT), epidermal cells (EPID), endothelial cells (ENDO), and smooth muscle cells (SMmus), which function prominently in the maintenance functions of wound healing and angiogenesis, are more densely connected than are the others; red blood cells (REDc) only receive one-way connections; and melanocytes (MELA) only put out one-way connections. Although the present databases for non-immune cells are likely to be incomplete, the body-cell cytokine architecture noted here indicates the high connectivity of the body cells involved in maintenance functions and suggests an important regulatory role for melanocytes [11]

### Immune and body connectivity

**Figure 4:**
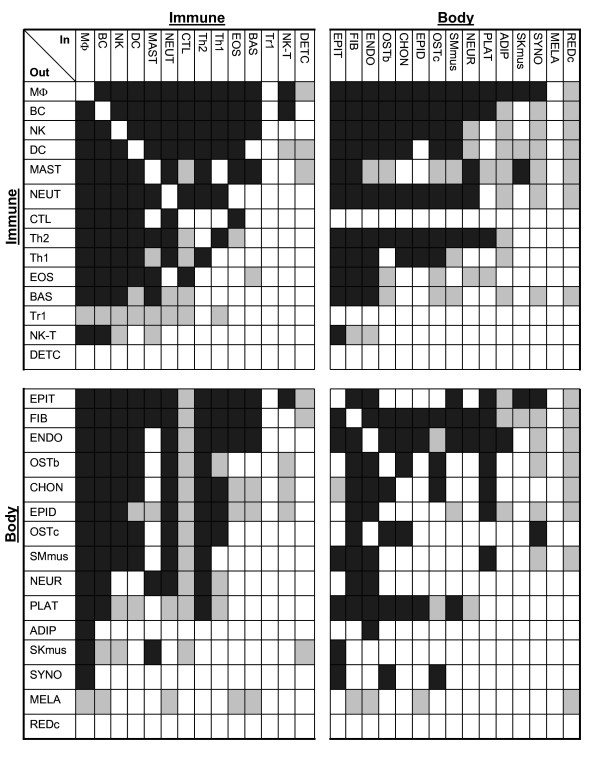
**Cytokine network connections between immune cells and body cells**. Mutual connections are coded as black squares and one-way connections as grey squares. A lack of a cytokine connection between the different cells (and self-connectivity) is coded by white squares.

Figure 4 illustrates the cell connections, mutual (Black squares) and one-way (Gray squares), of each of the 29 nodes – immune and non-immune body cells – in both input and output arrangements within and between each sub-network. It can be seen that the cell types most highly connected within the immune system (such as macrophages and B cells) are also the cell types most connected with body cells. Likewise, certain body cells manifest cytokine connections with many other cells. For example, epithelial cells, fibroblasts and endothelial cells are the body cells most highly connected to both immune and other body cells. The highly connected body cells are those that are prominent in the healing process[12]; this makes sense if indeed cytokine networks function in body maintenance[4,13].

### Social super-family

**Figure 5:**
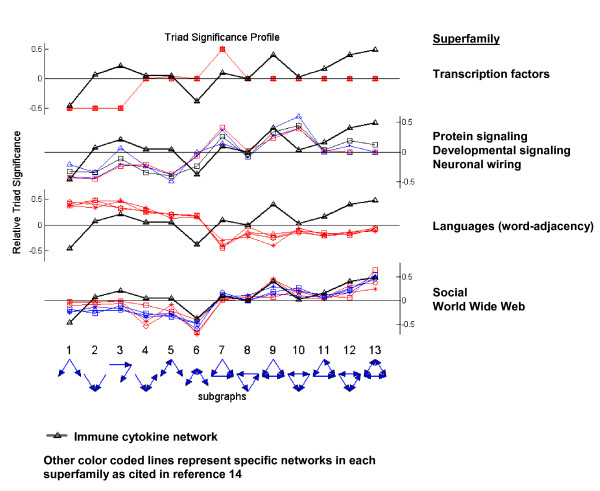
**Triad Significance Profile (TSP)**. Triad Significance Profile (TSP) of the immune-cell cytokine network superimposed on the TSP of four super-families (see ref 14 for details of the 4 super-families): transcription factor networks; protein and developmental signalling and neuronal wiring; languages (word adjacency); and human social networks and the World Wide Web. The TSP of the immune cytokine network fits only the fourth super-family.

Networks with common structural motifs can be grouped into super-families; super-families probably arise because networks with similar architectures have evolved to perform similar systems tasks[14]. One can identify a super-family by the motif profile of its three-cell networks; members of a super-family share motif profiles. We calculated the normalised significance level (Z score; see Methods) for each of the possible 13 triads (see Figure 5, bottom), as described[14], to derive the Triad Significance Profile (TSP). Figure 5 depicts the TSP of the immune cytokine sub-network superimposed on the TSP of four super-families (see reference 14 for details of the 4 super-families): transcription factor networks; protein and developmental signalling and neuronal wiring; languages (word adjacency); and human social networks and the World Wide Web. Inspection of Figure 5 reveals that the immune cytokine sub-network has a TSP most similar to the social and World Wide Web networks, especially in triad sub-graphs 6–13; only this super-family features the *clique *motif. The immune cell cytokine network TSP is clearly different from the other 3 super-families of molecular and linguistic networks.

## Discussion

Here we use an informatic methodology to uncover the large-scale architecture of the cytokine networks that connect immune and non-immune cells. The cytokine network architecture in this study (Figure 2, 3 and 5) emerged "bottom-up" from the published data gathered by the keepers of the two Internet databases we used. Thus, the data on which this study was based were not selected by us a priori to serve any theory or preconception about the immune system.

The present study, because of computational limitations, was limited to two-cell and three-cell structures; moreover, named cytokines and their individual effects could not be included in the analysis. Nevertheless, even this partial and static view of cytokine architecture calls attention to important features of immune systems biology. The clonal selection theory (CST) of adaptive immunity in its classical formulation proposed that the immune system functioned only to protect the body against foreign invaders, and was regulated entirely by the foreign antigens that happened to enter the body and activate independent clones of lymphocytes[15]. However, the dense and reciprocal cytokine architecture of immune and body cells disclosed here highlights the collective nature of immune behaviour; individual clones of lymphocytes are selected by specific antigens, but the biology of the immune response is regulated by the cytokines expressed by body cells and by the collective of interacting immune cells[4,13,16]. The high density and motif/anti-motif specificity of immune-body cytokine connectivity (Figure 1 and 4) are compatible with the idea that the immune system could be involved in the physiology of the body and not only in its defence [13,17]. It appears that particular network motifs can encode logic gates that generate specific functions[10,14,18]; further work is required to elucidate the logic-gate physiology of the particular cytokine motifs and anti-motifs disclosed here.

Note the high degree of cytokine connectivity between immune cells – macrophages, B cells, dendritic cells, and NK cells – and non-immune body cells – fibroblasts, endothelial cells, epithelial cells, platelets (PLAT), osteoblasts (OSTb), osteoclasts (OSTc), chondrocytes (CHON), and smooth muscle cells (Figure 4). Interestingly, these body cells are involved in wound healing[12], bone repair[19], blood-vessel formation[20], and other processes that maintain the healthy body[13]. At least from the cytokine point of view, the immune system and the body are closely connected to form a large integrated maintenance system[13].

A high density of internal networking is characteristic of cognitive systems such as the brain[8,13,21] – neurons spend much time communicating among themselves while they interact with their environment. Indeed, the high density of internal cytokine networking of immune cells (Figure 1 and Table 2) is compatible with the notion that the immune system may have evolved a process of cell integration that emerges from this collective exchange of cytokine signals[13,22]. The *clique *and *semi-clique *motifs are characteristic of immune system and social super-families (see Figure 5, triad sub-graphs 12 and 13) and of some neuron systems[8]. It would appear that mutual connectivity between immune cells is essential to integrated, collective immune function.

The cytokine architecture of the system as a whole extends and complements the known biologic roles of particular cells [1-3]. Figure 3a and 3b show us that the macrophage and the B cell are unique in being mutually connected to most other cells in the immune system, and also to many body cells. Interestingly, the macrophage and the B cell function as professional antigen-presenting cells [1-3]; T cells and B cells that bear receptors for specific antigens may respond to their antigens processed and presented by macrophages or B cells [1-3]. Thus, macrophages and B cells link antigen-specific immunity (antigen presentation) and innate immunity (reciprocal cytokine connectivity to immune and body cells) [1-3]. The macrophage is probably the first immune cell to appear in the evolutionary tree and the B cell is probably the first immune cell that bears a somatically generated receptor able to recognise antigens[23]. Thus a cell's position in the structure of the network might reflect its evolutionary position, and not only its function (Figure 3b). Extension of the cytokine network analysis to include individual cytokines will require increased computing and algorithmic power, but should reward us with a more precise understanding of the system.

Note that immune connectivity, unlike neuronal connectivity, is not hard-wired [1-3,24]. Since most cytokine interactions are between adjacent cells[4], the immune-body cytokine network is established by immune-cell migration to discrete body sites. The immune cytokine network materialises in practice only when immune cells migrate by chemotaxis to tissue sites of inflammation or to selected hubs of immune cell congregation – lymph nodes, Payer's patches, spleen and other immune organs [1-3,24]. The organised migration, compartmentalisation and selective activation of immune cells serve to channel the dense potential connectivity of the system into manageable, ad hoc collectives that gather and disperse, like human interactions, as the need arises[13]. Just as the architect's plan is realised only when living people interact in the standing structure, cytokine architecture is realised only when living cells interact dynamically in the living body.

## Conclusion

The cytokine connectivity architectures shown here demonstrate that immune cells do not function merely as individual clones, but work in innately integrated and hierarchical collectives. Indeed, the rich cytokine connectivity of immune cells with body cells involved in wound healing and angiogenesis is compatible with the concept that these cell collectives are integrated into a maintenance system[13]. Defense against pathogens is not the only function of the immune system.

## Methods

### Database: cells and cytokines

Table 1 lists the 29 nodes (14 immune cells and 15 non-immune cells) and 29 edges (major cytokines) we analysed [5-7]. We deleted redundancies and obvious errors from the databases, and grouped as one node any cell that appears in the database labelled in various states and locations (see additional files 1 and 2).

### Density

The density of a network [25] is the actual number of edges realised in the existing network as a fraction of the maximal number of edges potentially expressible in the network. The values are normalised, and range between 0 (no edges) and 1 (all possible edges exist). Network density is calculated thusly: *L *is the number of edges and *N *is the number of nodes in the network. In a directed edge network (the direction of the edges is recorded), the maximal number of edges for a network with *N *nodes is *N*(*N *- 1), and the density is *L*/*N*(*N *- 1). In an undirected network (the direction of the arrows is not considered), the maximal number of edges for *N *nodes is *N*(*N *- 1)/2, and the density is 2*L*/*N*(*N *- 1). Network density is the average fraction of edges incident with nodes in the network[25].

### Motifs, non-motifs and anti-motifs

The motif analysis[18] is made by comparing the *observed *frequency of a particular set of nodes and edges with the *estimated *frequency of the particular set. The estimated frequency of a set of connections is computed by first characterising the actual numbers of edges that enter (input) or exit (output) each node – these edges constitute the legitimate input and output of each node. Each node is then paired at random with other system nodes, but without changing the legitimate inputs and outputs of each node defined by the real system; see additional file 4.

A motif [18] is a particular pattern of connections – edges and nodes – that occurs in the actual network at an observed frequency significantly greater than the estimated frequency of the same pattern of connections obtained randomly.

A non-motif[18] is a pattern of edges and nodes with an observed frequency not significantly different than the expected, random frequency.

An anti-motif is defined here as a pattern of edges and nodes occurring in the real network at an observed frequency significantly less than the expected, random frequency of that pattern.

We used *wmfinder*, version 10.06 tool (available on request from [26]) to compare each observed Two-node or Three-node connections to their expected frequency obtained by examining 1000 randomised networks made by randomly switching the edges of the real network among the different nodes. Network motifs must meet the following criteria[18]: (i) The probability that the motif appears in a randomised network in an equal or greater number of times than in the real network is smaller than p = 0.01. The qualitative measure of statistical significance is the *Z *score = (*N*_observed _- *N*_estimated_)/SD). (ii) The number of times the motif appears in the real network with distinct sets of nodes is at least U = 2. (iii) The number of appearances in the real network is significantly larger than in the randomised networks: *N*_observed _- *N*_estimated _> 0.1*N*_estimated_. This avoids detecting as motifs common connections that differ only slightly between *N*_observed _and *N*_estimated_, but have a narrow distribution in the randomised networks.

Network anti-motifs meet the following criteria, as defined here: (i) The probability that the anti-motif appears in a real network in a frequency equal to or greater than the number of times it appears in the randomised network is less than p = 0.01. The qualitative measure of statistical significance is the *Z *score = (*N*_observed _- *N*_estimated_)/SD). (ii) The number of appearances in the real network is significantly lower than in the randomised networks: *N*_estimated _- *N*_observed _> 0.1*N*_observed_. This avoids detecting as anti-motifs common connections that only have a slight difference between *N*_observed _and *N*_estimated _but have a narrow distribution in the randomised networks.

## Reviewers' comments

### Reviewer's report 1

Neil Greenspan, MD, PhD, Professor of Pathology, Case Western Reserve University School of Medicine, Cleveland, OH, USA

This analysis, by Frankenstein and colleagues, of the cytokine networks among cells of the immune system, parenchymal cells in various tissues, and between immune and parenchymal cells brings to immunology a relatively abstract form of analysis that is worth exploiting more than it has been. It is reminiscent of the analyses of genetic networks and genome evolution by Stuart Kauffman. In Kauffman's models of genome evolution, for instance, loci are identified primarily on the basis of the number epistatic interactions and fitness values. Such 'stripped down' perspectives, devoid of the details most biologists routinely deal with, may feel alien to most experimentalists but they potentially facilitate the detection of large-scale patterns that may otherwise be difficult to discern. The study of Frankenstein *et al*. would appear to fulfil this promise in some measure by revealing interesting differences, as for example between different cellular subsets within the immune system, in overall patterns of cytokine connectivity. It is also worth noting that where Kaufmann's models were theoretical constructions, the present work is an analysis of actual data but without many of the molecular and cellular details that characterize the reporting of most immunological investigations.

Findings of particular interest were those relating to the different cytokine connectivity patterns for immune system cells (i.e., the identification of five tiers), such as B cells and macrophages vs. (for example) CD4+ or CD8+ T cells. Also of interest were the global differences in connectivity patterns for immune cells with one another vs. parenchymal cells with one another and the relatively high densities of cytokine connectivity in the immune and parenchymal cell networks. While some of the findings could be claimed to have been already apparent in some degree or respect, others are unlikely to have been noticed amidst the thicket of information reported in a typical experimental report. For example, it caught my eye that immune cell types that interacted with a given type of body cell are very likely to interact with each other as well.

My issues with some of the wording in the opening paragraph of the abstract have been satisfactorily resolved. In addition, my suggestion that the authors include classical adaptive immune responses along with inflammation and healing as processes involving cytokine-associated signals has been addressed.

The issue of cytokine networks being "innately connected" deserves further comment. In one sense it is definitely true that cytokine networks are innate. Which cells secrete and which cells have receptors for and respond to given cytokines are determined (at least in large part) by information inherited through the germline. (The parenthetical statement just leaves room for the possibility that environmental variations might exert some influence on this aspect of the organismal phenotype.) So in this sense, cytokine networks are innate.

The problem with using this sense of "innate" in the context of distinguishing between "innate immunity" and "adaptive immunity" is that almost every aspect of the adaptive immune response, except for the final products of the immunoglobulin (Ig) and T cell receptor (TCR) gene rearrangements, are similarly determined (at least in large part) by information inherited through the germline. After all, the enzymes that orchestrate the Ig and TCR gene rearrangements are germline encoded and are expressed without obvious regard for any particular antigens. The signaling pathways emanating from the antigen-specific receptors on B and T lymphocytes, and those associated with the various co-stimulatory receptors, are likewise determined (at least in large part) by information inherited through the germline. Similarly, the co-stimulatory receptors (e.g., CD21 on B cells and CD4 or CD8 on T cells) are encoded by germline genes that are normally unaltered by the clonally-varying Ig or TCR gene rearrangements. My point is that while the terms "innate immunity" and "adaptive immunity" are widely employed, the process of thinking through what precisely these terms mean is less-widely employed.

#### Author's response

*Cytokine connections are innate; they interact with innate receptors. however, the revision accepts your point; we do not intend to slight the adaptive arm of the immune response*.

Some questions regarding the analysis follow:

How robust are the identified patterns? Will they hold up in further analyses, when, for example, the motifs studied can involve more than three cell types at one time or when individual cytokines are identified?

#### Author's response

*The statistical analysis done here suggests that the motifs are robust. Your questions are certainly valid, but await more computational power – hopefully in the near future*.

Will the discovery of new cytokines (or the inclusion of already discovered but not yet included cytokines, such as IL-23) influence the various cytokine connectivity patterns identified so far? What about chemokines?

#### Author's response

*Good questions; the future will tell. The present study should be viewed as an opener*.

Related to these questions, do analyses performed separately on the two databases, in those areas where they overlap (if they do), yield comparable results?

#### Author's response

*The data bases largely overlap, so we could combine them. Biologically questionable data, as explained in the paper, were removed before analysis*.

Would it be valuable to take into account quantitative aspects of cytokine secretion (if it is experimentally feasible)?

#### Author's response

*This is not yet feasible technically*.

Is there any possibility that higher-resolution distinctions among different types of cells, say B or T cells, for instance, would alter the apparent connectivity patterns [e.g., what was considered a two-way interaction between atype of B (T) cells and another, let us say non-immune, cell type actually involves a signal sent from one B (T) cell subset and a signal received by a 'different' B (T) cell subset, transforming one mutual interaction into two one-way interactions]?

Is it possible that genetic polymorphisms will influence cytokine connectivity sufficiently to generate more than one real pattern within a species?

#### Author's response

*There are not enough data available yet to do that*.

If cytokine connectivities are context-dependent (where context very much includes the full range and quantities of cytokines in a local environment), is it fair to display the connectivities in static network diagrams?

#### Author's response

*The static view shows us the conduits available for dynamic interactions, to be studied when the data and the computational power permit*.

##### Conclusion

Overall, I think this study identifies interesting trends and should stimulate immunologists to think about immunity and the immune system in new ways. The extensive cytokine connections between cells of and not of the immune system, at least as it is classically defined, should provoke useful reflection on the possible limits of conceptualizing the immune system narrowly and in isolation from other physiological systems. Finally, I note that the acknowledgments by the authors of the necessary limitations of this beginning effort to explore the large-scale patterns of cytokine connectivity and the value of using different scales of resolution in future analyses enhance my appreciation for this study.

### Reviewer's report 2

Matthias von Herrath, MD, Professor and Head, Immune Regulation laboratory, La Jolla Institute for Allergy and Immunology, San Diego, CA, USA

The paper is now much clearer as to the methodology behind the work.

Now corrected I understand that this is the currently best algorithm for the task and it doesn't allow the names of the cytokines to be known. The paper or follow-up studies should have a strong impact, once new algorithms have been created that can show the whole picture.

The additional material does allow the user to browse the data themselves in a very inaccessible manner. It would be very helpful to biologists to have an easily browse-able webpage or a self-extracting executable.

#### Author's response

Additional file 2 – ***Supplementary_Data.doc ****is intended to satisfy this need*.

While I agree with the author that the table 2 does require verbal explanation I still find the majority of the explanation a bit long.

Pls clearly explain 'motif' on page 4, if possible.

#### Author's response

*The Methods section and the references cited there should clarify the subject*.

### Reviewer's report 3

Professor Anne Cooke, Department of Pathology, University of Cambridge, Cambridge CB21QP, UK

This manuscript describes the findings of an informatic approach to describe the network of cytokine interactions that are involved in communications between the immune system and body cells and that are intimately involved in maintaining body integrity. The basic information utilised in this analysis was derived from cytokine databases. Work has already been published modelling the immune system interactions with its mediators that has demonstrated hierarchies within mediators of network relevance. This manuscript addresses the extra complexity introduced by adding body cell interactions and their interface with the immune system and mediators into the analysis. Interesting properties emerge from this kind of analysis, in particular, the mutual connectivities between immune cells and between the immune system and the body.

Of course, there are always going to be concerns about how much will the system described be perturbed by the discovery of new cytokines, new cells (eg Th17) or increased characterisation of cell types eg T reg. The nervous system did not seem to be feature in this analysis and much emphasis was placed on wound healing and angiogenesis. It is also sometimes interesting to consider normal processes which are involved in tissue remodelling such as mammary gland involution which clearly involves cytokines as well as cells of the innate immune system. Nevertheless, this manuscript represents a useful approach. The key test of its robustness will be how it accommodates new mediators and cell types and their impact on the other cells in these networks.

## Abbreviations

Cytokines:

IFN-Interferon

IL-Interleukin

CSF-Colony Stimulating Factor

TGF-Transforming Growth Factor

TNF-Tumor Necrosis Factor

MIF-Migration Inhibition Factor

Immune cells:

MΦ-Macrophage/Monocyte

NK-T-Natural Killer T cell

NK-Natural Killer cell

DC-Dendritic cell

Th1-T helper 1

EOS-Eosinophil

Th2-T helper 2

BAS-Basophile

CTL-Cytotoxic T Cell

NEUT-Neutrophile

Tr1-T Regulatory 1

BC-B cell

DETC-Dendritic Epidermal T cell

MAST-Mast cell

Body cells:

FIB-Fibroblast

OSTb-Osteoblast

EPIT-Epithelial cell

OSTc-Osteoclast

ENDO-Endothelial cell

ADIP-Adipocyte

PLAT-Platelet

SYNO-Synovial cell

CHON-Chondrocyte

REDc-Red Blood cell

NEUR-Neuronal cell

EPID-Epidermal cell

SMmus-Smooth muscle cell

MELA-Melanocyte

SKmus-Skeletal muscle cell

## Competing interests

The author(s) declare that they have no competing interests.

## Authors' contributions

ZF performed the study; he collected the data, ran the algorithms, contributed much to the interpretations, and created the figures. UA and IRC supervised the work; UA supplied the informatics background and the algorithms and IRC provided the immunology and contributed the biologic interpretations; IRC also wrote the text of the paper.

## Supplementary Material

Additional File 1 Supplementary Method. This is a detailed description of the cytokine network creation. Click here for file

Additional File 2 Supplementary Data. This is a description for the cytokine network txt files (additional files 5, 6, 7, 8, 9), which includes the detailed collected cell interactions. Click here for file

Additional File 3 Supplementary Density Table. This table shows the densities of 113 published networks. Density was determined by the published number of nodes, N, and edges, L (see article Methods) for each network. For each network we show the number of nodes, N, number of edges, L, density values and the reference. Click here for file

Additional File 4 Supplementary Network Randomisation Rules Figure. The figure shows the network randomisation rules. Click here for file

Additional File 5 Cytokine Errors. Text file that includes all the interactions that were obvious errors in the database (see Additional file 2). Click here for file

Additional File 6 Global Cytokine Interactions. Text file that includes all the interactions in the network (see Additional file 2). Click here for file

Additional File 7 Immune Cytokine Interactions. Text file that includes the immune cells interactions in the immune sub-network (see Additional file 2). Click here for file

Additional File 8 Body Cytokine Interactions. Text file that includes the body cells interactions in the immune body sub-network (see Additional file 2). Click here for file

Additional File 9 Interface Cytokine Interactions. Text file that includes the immune to body cells and body to immune cells interactions in the sub-network (see Additional file 2). Click here for file
