# Supplementary material for: The immune-body cytokine network defines a social architecture of cell interactions
Source: Biol Direct. 2006 Oct 24;1:32. doi: 10.1186/1745-6150-1-32 (PMC1636025; doi:10.1186/1745-6150-1-32)
Supplement: Additional File 1 — Supplementary Method. This is a detailed description of the cytokine network creation. [file 1745-6150-1-32-S1.doc]

**Supplementary Method**

**Network creation**

Cytokine connections between immune and body cells were obtained manually from two Internet databases: the Cytokines Online Pathfinder Encyclopedia (COPE)[1, 2] and the Cytokine Reference - Online Database[3]. We transformed automatically the raw data into a network format designating cells as nodes and cytokine connections as edges. We neglect problematic cells; for example, abnormal cells (leukæmia, glioblastoma, oligodendroglioma etc), problematic cells (lymphoblast, Burst Forming Unit Erythroid, etc), too generalized (T-lymphocyte, hæmatopoietic cells, granulocytes etc), or progenitors (pre-B-cell, monoblast, immature dendritic cells etc). We also deleted from the analysis immobile body cells that were restricted to certain organs (ovarian granulose, pituitary, leydig etc), and would therefore not be expected to be able to interact directly with other anatomically restricted cells. The body-cell interactions deleted from the analysis are listed in additional files 2 and 5. The standard immune and body cells (Table 1) so obtained and were each grouped as one cell although that cell might appear in the database labelled in various states such as “resting”, “activated”, “naïve”, and in various locations as "peritonæal", "peripheral", "tissue", etc. This reduction in cells resulted in 2461 cytokine interactions connecting 29 nodes (immune and non-immune system cells) by 29 major cytokines (G–CSF, GM–CSF, IL-1, IL-2, IL-3, IL-4, IL-5, IL-6, IL-7, IL-8, IL–9, IL-10, IL–11, IL-12, IL-13, IL-15, IL–16, IL-18, IL-22, IL–27, M–CSF, MIF, IFN–alpha, IFN–beta, IFN-gamma, IFN-kappa, TGF–beta, TNF–alpha, TNF-beta). We further reduced the redundancy of cytokine connections in the databases by recording only one edge between any two nodes, irrespective of how many different cytokine signals the two cells might exchange; this procedure reduced 2461 cytokine interactions to 455 edges. We further deleted 37 connections that were obvious errors (see additional file 2). This left 418 edges and 29 nodes in the global network (see additional file 2).

**Table 1 - Cells (nodes) in the analysis**

| Immune cells  (nodes) | MΦ- Macrophage/Monocyte |
| --- | --- |
|  | NK- Natural Killer cell |
|  | Th1- T helper 1 |
|  | Th2- T helper 2 |
|  | CTL- Cytotoxic T Cell |
|  | Tr1- T Regulatory 1 |
|  | DETC- Dendritic Epidermal T cell |
|  | NK-T- Natural Killer T cell |
|  | DC- Dendritic cell |
|  | EOS- Eosinophil |
|  | BAS- Basophile |
|  | NEUT- Neutrophile |
|  | BC- B cell |
|  | MAST- Mast cell |
| Body cells  (nodes) | FIB- Fibroblast |
|  | EPIT- Epithelial cell |
|  | ENDO- Endothelial cell |
|  | PLAT- Platelet |
|  | CHON- Chondrocyte |
|  | NEUR- Neuronal cell |
|  | SMmus- Smooth muscle cell |
|  | SKmus- Skeletal muscle cell |
|  | OSTb- Osteoblast |
|  | OSTc- Osteoclast |
|  | ADIP- Adipocyte |
|  | SYNO- Synovial cell |
|  | REDc- Red Blood cell |
|  | EPID- Epidermal cell |
|  | MELA- Melanocyte |

**References**
