# Supplementary material for: The immune-body cytokine network defines a social architecture of cell interactions
Source: Biol Direct. 2006 Oct 24;1:32. doi: 10.1186/1745-6150-1-32 (PMC1636025; doi:10.1186/1745-6150-1-32)
Supplement: Additional File 2 — Supplementary Data. This is a description for the cytokine network txt files (additional files 5, 6, 7, 8, 9), which includes the detailed collected cell interactions. [file 1745-6150-1-32-S2.doc]

**Supplementary Data**

**Data files for the cytokine network**

CytokineErrors _ts.txt (Additional file 5) is a text file that includes all the body-cell interactions that were deleted from the analysis because they were unlikely to occur anatomically. The convention used in the file is that the order of the columns is: cytokine target cell, cytokine source cell. The conversion between node indexes and cell names can be found in Table 1.

GlobalCytokineInter_ts.txt (Additional file 6) is a text file that includes all the interactions in the network. The convention used in the file is that the order of the columns is: cytokine target cell, cytokine source cell, mode of connection.

In the GlobalCytokineInter_ts.txt network the mode of connection is:

1 = Immune cell connection

2 = Body cell connection

3 = Body to Immune cell connection

4 = Immune to Body cell connection

The conversion between node indexes and cell names can be found in Table 1.

ImmuneCytokineInter_ts.txt (Additional file 7) and BodyCytokineInter_ts.txt (Additional file 8) are text files that include the immune and body cell interactions, respectively, in the sub-networks. The convention used in the file is that the order of the columns is: cytokine target cell, cytokine source cell, mode of connection. The basis of the mode of connection is always 1. The conversion between node indexes and cell names can be found in Table 1.

InterfaceCytokineInter_ts.txt (Additional file 9) is a text file that includes the immune to body cell and body to immune cell interactions in the sub-network. The convention used in the file is that the order of the columns is: cytokine target cell, cytokine source cell, mode of connection. The basis of the mode of connection is always 1. The conversion between node indexes and cells names can be found in Table 1.

**Table 1 - Conversion between node indexes and cell names**

| Immune cells  (nodes) | **Index** | **Abbreviation- Cell name** |
| --- | --- | --- |
|  | 2 | MΦ- Macrophage/Monocyte |
|  | 8 | NK- Natural Killer cell |
|  | 17 | BC- B cell |
|  | 22 | NEUT- Neutrophile |
|  | 83 | Th1- T helper 1 |
|  | 84 | Th2- T helper 2 |
|  | 85 | CTL- Cytotoxic T Cell |
|  | 88 | MAST- Mast cell |
|  | 91 | EOS- Eosinophil |
|  | 93 | BAS- Basophile |
|  | 146 | DC- Dendritic cell |
|  | 191 | Tr1- T Regulatory 1 |
|  | 199 | DETC- Dendritic Epidermal T cell |
|  | 235 | NK-T- Natural Killer T cell |
| Body cells  (nodes) | 4 | FIB- Fibroblast |
|  | 5 | EPIT- Epithelial cell |
|  | 11 | ENDO- Endothelial cell |
|  | 25 | PLAT- Platelet |
|  | 28 | CHON- Chondrocyte |
|  | 31 | NEUR- Neuronal cell |
|  | 37 | SMmus- Smooth muscle cell |
|  | 39 | OSTb- Osteoblast |
|  | 52 | OSTc- Osteoclast |
|  | 53 | ADIP- Adipocyte |
|  | 54 | SYNO- Synovial cell |
|  | 177 | EPID- Epidermal cell |
|  | 219 | REDc- Red Blood cell |
|  | 281 | SKmus- Skeletal muscle cell |
|  | 285 | MELA- Melanocyte |
