# Supplementary material for: The immune-body cytokine network defines a social architecture of cell interactions
Source: Biol Direct. 2006 Oct 24;1:32. doi: 10.1186/1745-6150-1-32 (PMC1636025; doi:10.1186/1745-6150-1-32)
Supplement: Additional File 3 — Supplementary Density Table. This table shows the densities of 113 published networks. Density was determined by the published number of nodes, N, and edges, L (see article Methods) for each network. For each network we show the number of nodes, N, number of edges, L, density values and the reference. [file 1745-6150-1-32-S3.doc]

**Supplementary Density Table**

Densities of 113 published networks. Density was determined by the published number of nodes, N, and edges, L (see Methods) for each network.

* Undirected network

- This paper

**References**

1. Milo R, Itzkovitz S, Kashtan N, Levitt R, Shen-Orr S, Ayzenshtat I, Sheffer M, Alon U: **Superfamilies of evolved and designed networks**. *Science* 2004, **303**:1538-1542.

2. Sporns O, Kotter R: **Motifs in brain networks**. *PLoS Biol* 2004, **2**:e369.

3. Giot L, Bader JS, Brouwer C, Chaudhuri A, Kuang B, Li Y, Hao YL, Ooi CE, Godwin B, Vitols E, Vijayadamodar G, Pochart P, Machineni H, Welsh M, Kong Y, Zerhusen B, Malcolm R, Varrone Z, Collis A, Minto M, Burgess S, McDaniel L, Stimpson E, Spriggs F, Williams J, Neurath K, Ioime N, Agee M, Voss E, Furtak K, Renzulli R, Aanensen N, Carrolla S, Bickelhaupt E, Lazovatsky Y, DaSilva A, Zhong J, Stanyon CA, Finley RL, Jr., White KP, Braverman M, Jarvie T, Gold S, Leach M, Knight J, Shimkets RA, McKenna MP, Chant J, Rothberg JM: **A protein interaction map of Drosophila melanogaster**. *Science* 2003, **302**:1727-1736.

4. Przulj N, Wigle DA, Jurisica I: **Functional topology in a network of protein interactions**. *Bioinformatics* 2004, **20**:340-348.

5. Lehner B, Fraser AG: **A first-draft human protein-interaction map**. *Genome Biol* 2004, **5**:R63.

6. Jeong H, Tombor B, Albert R, Oltvai ZN, Barabasi AL: **The large-scale organization of metabolic networks**. *Nature* 2000, **407**:651-654.

7. Newman MEJ: **The Structure and Function of Complex Networks**. *SIAM Review* 2003, **45**:167-256.

8. Li W, Cai X: **Statistical analysis of airport network of China**. *Phys Rev E Stat Nonlin Soft Matter Phys* 2004, **69**:046106.
