# Supplementary material for: The immune-body cytokine network defines a social architecture of cell interactions
Source: Biol Direct. 2006 Oct 24;1:32. doi: 10.1186/1745-6150-1-32 (PMC1636025; doi:10.1186/1745-6150-1-32)
Supplement: Additional File 4 — Supplementary Network Randomisation Rules Figure. The figure shows the network randomisation rules. [file 1745-6150-1-32-S4.ppt]

## Slide 1
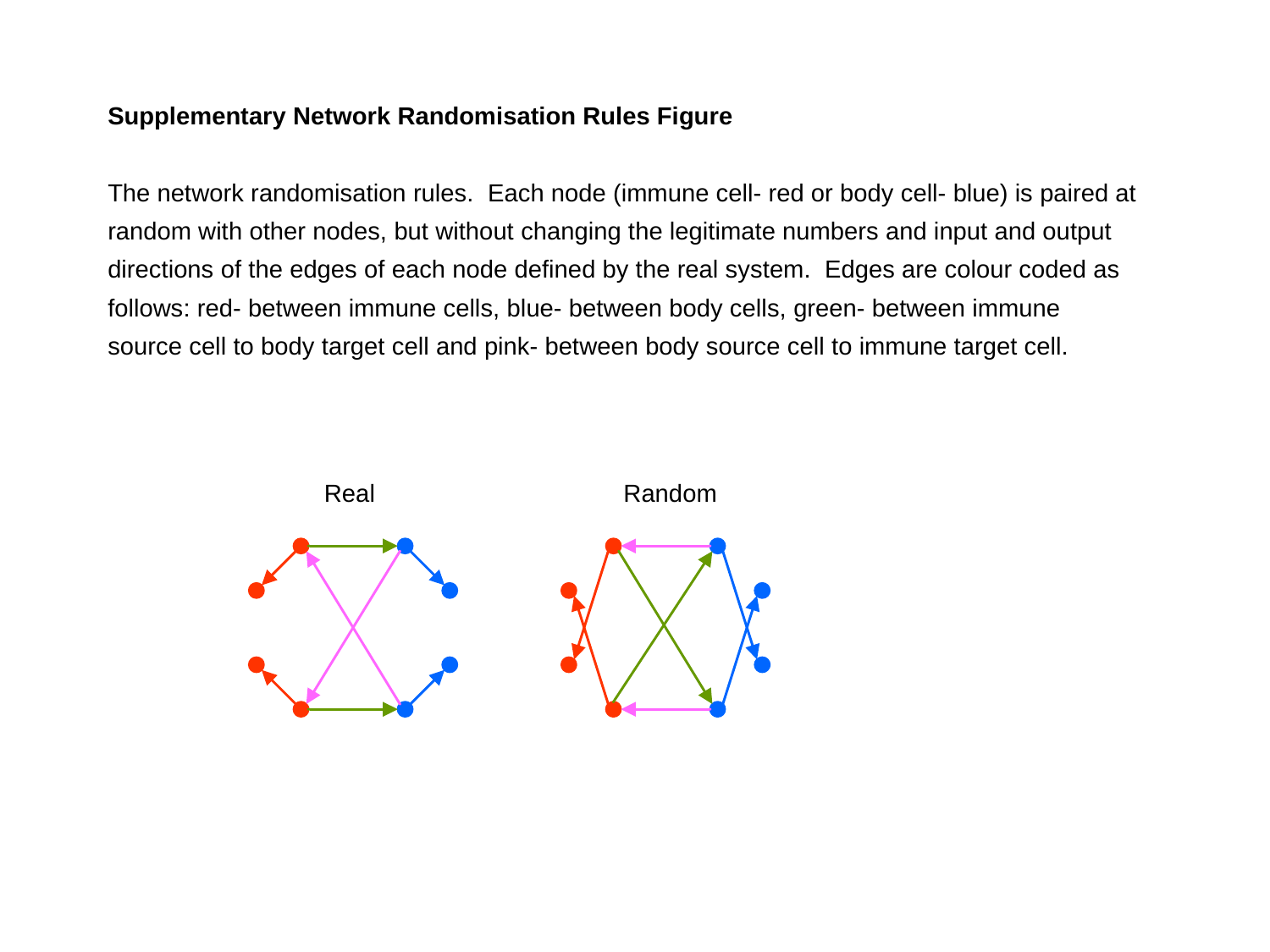

Supplementary Network Randomisation Rules Figure
The network randomisation rules. Each node (immune cell- red or body cell- blue) is paired at random with other nodes, but without changing the legitimate numbers and input and output directions of the edges of each node defined by the real system. Edges are colour coded as follows: red- between immune cells, blue- between body cells, green- between immune source cell to body target cell and pink- between body source cell to immune target cell.
